# Genetic and epigenetic analyses of *MBD3* in colon and lung cancer

**DOI:** 10.1038/sj.bjc.6601776

**Published:** 2004-04-13

**Authors:** Y Zhu, D J Harrison, S A Bader

**Affiliations:** 1 Sir Alastair Currie Cancer Research UK Laboratories, Division of Pathology, Molecular Medicine Centre, University of Edinburgh, Western General Hospital, Crewe Road, Edinburgh EH4 2XU, UK

**Keywords:** MBD3, colon cancer, lung cancer, mutation, methylation

## Abstract

*MBD3* is a member of the methyl-CpG-binding domain family and is located on chromosome 19p13.3, a region of loss of heterozygosity in colon and lung cancers. We therefore screened samples for abnormalities in *MBD3*. Our results indicate that *MBD3* is not a major target of genetic and epigenetic alteration in these cancers.

In humans, the somatic genome is globally methylated, with the exception of CpG islands. CpG islands are CG-rich regions of DNA, which are coincident with the promoters of 60% of human housekeeping genes (Antequera and Bird, 1994). DNA methylation is an important epigenetic modification in the human genome, playing an essential role in the control of gene expression. It is well established that DNA methylation is extensively involved in gene imprinting, X inactivation and development. Aberrant methylation of CpG islands in the promoter of many cancer-related genes results in silencing of their expression compared with normal cells of the same tissue (Tycko, 2000).

The signal encoded by a particular pattern of DNA methylation is transduced through proteins that bind to methylated CpGs. These proteins contain a specific domain, the methyl-CpG-binding domain (MBD) (Nan *et al*, 1993). So far, five human MBD proteins have been identified as members of this protein family: MBD1, MBD2, MBD3, MBD4 and MECP2. MBD3 shares about 70% of overall identity with MBD2 over most of their length (Hendrich and Bird, 1998). MBD3 is a subunit of the NuRD complex that has nucleosome remodelling and histone deacetylase activities (Wade *et al*, 1998,^1999^; Zhang *et al*, 1999). Although MBD3 does not itself bind directly to methylated DNA, several lines of evidence suggest that MBD3 does play a role as part of the NuRD complex in the maintenance of transcriptionally repressed chromatin. MBD3 is crucial to normal mammalian development as knockout of *Mbd3* in mice is embryonic lethal (Hendrich *et al*, 2001). Mutations or abnormal expression of *MBD3* could therefore play a role in tumorigenesis via inappropriate regulation of other gene expression. Other members (*MBD2* and *MBD4*) of the MBD family have already been associated with colorectal cancer, albeit in different mechanisms. Maintenance of *MBD2* expression (normal function being the suppression of transcription from methylated DNA) is required for tumour formation in *APC*^*min*^ mice (Sansom *et al*, 2003). Mutation of *MBD4* (normal function being the repair of methyl-CpG deamination events) occurs in microsatellite unstable tumours (Bader *et al*, 1999) and absence of the gene in MBD4 null mice leads to increased genomic mutations and tumour burden in *APC*^*min*^ mice (Millar *et al*, 2002).

*MBD3* is located on chromosome 19p13.3, a region reported to suffer 20–50% loss of heterozygosity (LOH) in sporadic colorectal carcinomas (Dong *et al*, 1998; Resta *et al*, 1998; Trojan *et al*, 2000). According to the data compiled by the Human Genome Mapping Project (available on the website http://www.ncbi.nlm.nih.gov/), *MBD3* is within about 500 kb of the gene *LKB1/STK11*, which is mutated or abnormally methylated in Peutz–Jeghers syndrome. Peutz–Jeghers patients have hamartomatous polyposis of the gastrointestinal tract and an increased risk of a range of cancers including colon. *LKB1* is rarely mutated or methylated (maximum about 20%), however, in sporadic colorectal carcinomas (Avizienyte *et al*, 1998; Resta *et al*, 1998; Esteller *et al*, 2000; Launonen *et al*, 2000; Trojan *et al*, 2000), raising the possibility that another gene in the vicinity is involved in these cancers. The short arm of chromosome 19 is also implicated in up to 86% of lung cancers (Lukeis *et al*, 1990; Virmani *et al*, 1998; Sanchez-Cespedes *et al*, 2001). In the light of the location of *MBD3* in a region of chromosomal loss and its known functions in transcription suppression, it is considered as a candidate tumour-suppressor gene. We therefore performed a mutation screen, expression study and methylation status assay to investigate the possible role of *MBD3* in the aetiology of colon and lung cancers.

## MATERIAL AND METHODS

### Samples, DNA and RNA extractions

Cancer cell lines were obtained from the European Collection of Cell Cultures (ECACC)/ATCC, comprising seven colon cancers, 20 lung cancers and one normal lung cell (BW1799) (see Table 1

**Table 1:** Cell lines and primers used

**Cell lines tissue**	**Description**	**Name**
Colon	Microsatellite stable	HT29, SW480, COLO320
	Microsatellite unstable	HCT116, DLD1/HCT15, LOVO,LS180
Lung	Small cell (SCLC)	NCI-H69, -H524, -H740, -H1672, -H1092
		-H1184, -H1838, COR-L24, -L47, -L51, -L88, -L279,-L311
	Non samll cell (NSCLC)	NCI-H358, -H835, -H920, -H1648, -H2122, COR-L23, -L105
		
**Primers exon**	**Primer name and sequence**	**PCR conditions per pair**
1	17: ACTGGCAGCTCGCAAGGCACA	95 3′, (95 30″, 55 30″, 68 2′) × 35
	15: GCACGCACGCACGACGCA	68 3′, Pfx pdymerase+5x Enhancer
2	5: TGGGTTTGGGGTCTTGGGGT	94 3′ (94 30′, 55 30″) × 35, 72 3′
	6: GTCACCTGCGTGACGCCA	Taq polymerase+10% DMSO
3	7: CAGGCCGGACTGCATATC	94 3′, (94 30″, 55 30″) × 35, 72 3′
	8: TTGGGGTCGCTGTGCGTT	Taq polymerase
4	9: GGGCCACTCTTGAGGTTCACA	94 3′, (94 30″, 58 30″) × 35, 72 3′
	10: TCCGCCTCCCCTCAGGGA	Taq polymerase
5	11: CTTGCGGCTGTTTGTCCA	94 3′, (94 30″, 58 30″) × 35, 72 3′
	12: TGGAGCAGCAGGGGACCA	Taq polymerase+5% DMSO
6	13: TGGTGTTAACGCAAGGTCCA	94 3′, (94 30″, 58 30″) × 35, 72 3′
	4: CACTGCCAGGACCCGACT	Taq polymerase+5% DMSO

). In all, 51 primary colon tumour samples were part of an unselected, anonymised collection from patients at the Royal Infirmary Edinburgh. DNAs were extracted by standard methods from pellets of cell lines, or from frozen primary tumours. Total RNAs of cell lines were extracted by using Trizol Reagent (Invitrogen) according to the manufacturer's protocol; normal colon tissue RNA was from Stratagene. PolyA+ RNA was isolated using a Qiagen direct mRNA kit according to the manufacturer's protocol. A measure of 10 *μ*g of polyA+ RNA was electrophoresed on a 7% formaldehyde gel and then transferred to Hybond-N (Amersham) nylon membrane by capillary transfer. The membrane was crosslinked by UV light before hybridisation. The northern blot was pre-hybridised for 30 min at 68°C with ExpressHyb solution (Clontech) and hybridised for 1 h at 68°C in the same solution with 50 *μ*Ci ^32^P-labelled MBD3 cDNA probe. The cDNA probe was PCR amplified by primer pair MBD3/3, 5′-ACATGCTGGGGGACGTGGA, and MBD3/30, 5′-GCTGCACAGTGGGTGATGTGA, which generated a 587 bp fragment. After appropriate washings, the membrane was exposed to Kodak XAR5 film at −70°C for up to 3 days. Autoradiographic exposure of a low specific activity (so as not to obscure the nearby 2.4 kb *MBD3* band) *β*-actin probe was used as a loading control for the lanes.

### Examination of MBD3 expression by RT–PCR and Northern blot hybridisation analyses

Total RNA (10 *μ*g) was reverse transcribed with random hexamers using the M-MLV Reverse Transcriptase kit (Invitrogen) according to the manufacturer's instructions. The cDNA (0.5 *μ*l) was then amplified in a 20 *μ*l reaction under standard conditions. The PCR cycle parameters were as follows: an initial denaturing step at 95°C for 5 min, followed by 35 cycles of (30 s at 95°C, 30 s at 52°C, 30 s at 72°C), then 3 min at 72°C. The primers used were MBD3/1, 5′-GAAGAAGTTCCGCAGCAA, and MBD3/2a, 5′-GGTCGCTCTTGACCTTGT, which generated a fragment of 260 bp. The PCR products were run on 1% agarose gel in 1 × TAE, and visualised by ethidium bromide staining.

### CpG island methylation status analysis

**Figure 1:**
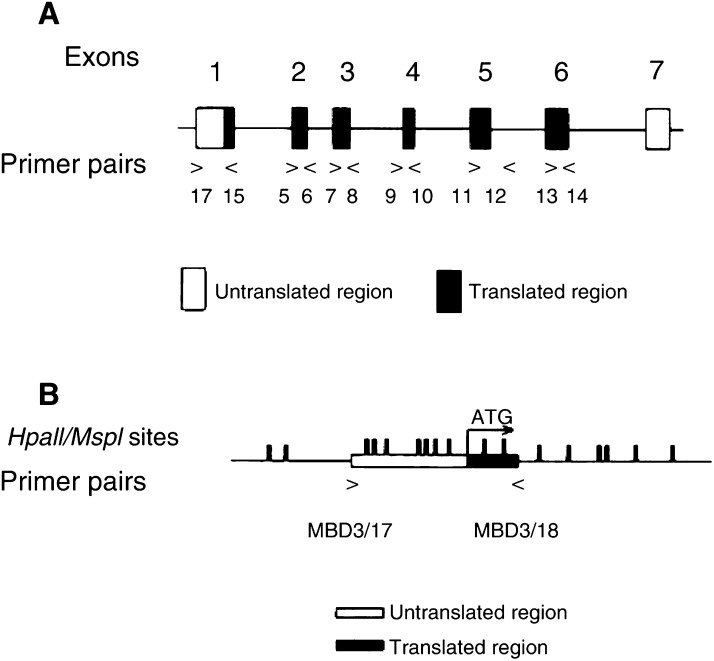
(**A**) *MBD3* gene structure. (**B**) *MBD3* CpG island *Hpa*II*/Msp*I restriction sites (CCGG), from Genbank AC005943.

To examine the methylation status of the *MBD3* CpG island, methylation-sensitive/resistant enzymes (*Hpa*II*/Msp*I) were used to digest DNA at CCGG sites, followed by PCR amplification. The presence of the PCR product from *Hpa*II digest indicates that the DNA is protected by methylation from being cut, while no PCR product results if the DNA is not methylated. No PCR product will be obtained from *Msp*I digested DNA because it cuts irrespective of its methylation status. Genomic DNA (0.5 *μ*g) from cancer cell lines was digested with 50 U of either *Hpa*II or *Msp*I (NEB) for 16 h at 37°C. The same amounts of fully methylated DNA (Sigma) were also digested as positive controls. The digest reactions were phenol : chloroform extracted and ethanol precipitated. Digested DNAs were then amplified by PCR using primer pairs MBD3/17, 5′-ACTGGCAGCTCGCAAGGCACA, and MBD3/18, 5′-CGCTGGGAGGAGCCCGTTGAG, which cover 531 bp within the CpG islands in exon 1(see Figure 1

). We used Platinum *Pfx* DNA polymerase (Invitrogen) to amplify the CG-rich region. Each 50 *μ*l reaction contained 1 × Pfx buffer, 0.3 M dNTP, 1 mM MgSO2, 0.3 *μ*M primers, 5 × PCR enhancer solution and 2.5 U *Pfx* DNA polymerase. The PCR conditions were 5 min at 97°C as a hot start, then 5 min at 95°C followed by 35 cycles of (30 s at 95°C, 30 s at 55°C, 2 min at 68°C) and finally 5 min at 68°C. The PCR products were run on 1% agarose gels and visualised by ethidium bromide staining.

### Mutational analysis by SSCP and sequencing

PCR of genomic DNA was carried out using six pairs of intron primers as listed in Table 1. These primer sets covered the entire coding region (exons 1–6) of *MBD3* and included splice acceptor and donor sites (see Figure 1). PCR conditions are included in Table 1. In all, 1 *μ*Ci of ^33^P-dATP (ICN) was added in each 10 ml reaction. PCR products were denatured and run on 0.5 × SequaGel MD gels (National Diagnostics) containing 10% glycerol and 0.6 × TBE for 18 h at 6W at room temperature. To identify base changes, the PCR products showing aberrantly migrated bands on SSCP gels were sequenced in both strands using the Thermo Sequenase Radiolabelled Terminator Cycle Sequencing Kit (USB) according to the manufacturer's protocol.

## RESULTS

**Figure 2:**
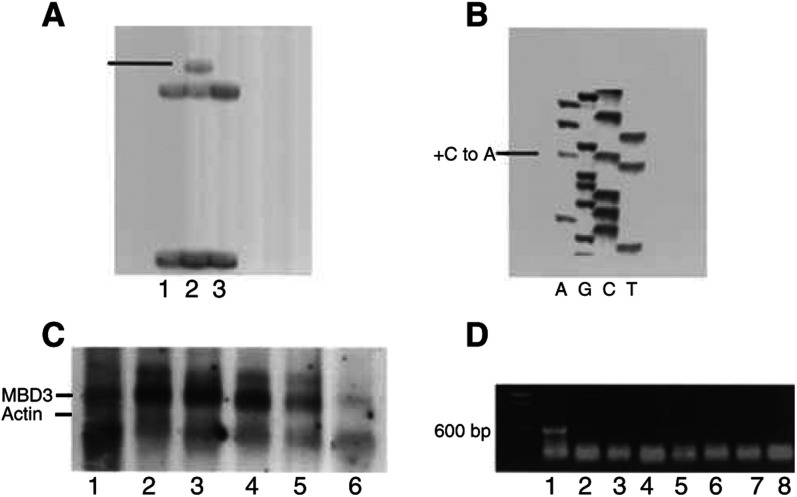
(**A**) Exon 6 abnormal bandshift on SSCP gel. Cell lines 1, 2 and 3 are HCT116, DLD1 and LOVO, respectively. (**B**) Heterozygous base change in exon 6 of DLD1. (**C**) *MBD3* polyA+ Northern hybridisation. Lanes 1–6 are: SW480, COLO320, HCT116, LOVO, LS180 and total RNA of the normal colon tissue, respectively. (**D**) Methylation-sensitive *Hpa*II restriction followed by PCR. Lanes 1–9 are: 100 bp marker, methylated positive control, HT29, SW480, Colo320, HCT116, DLD1, HCT15 and LOVO, respectively.

We found three aberrant SSCP bandshifts in 28 cell lines and 51 primary tumour samples, which were then sequenced (Figure 2A, B

). All of the changes were heterozygous, as seen by the retention of normal bands in SSCP or sequencing gels, and all were in cell lines. Specifically, in exon 3 of DLD1/HCT15 (cell lines derived from the same tumour), a G to A transition in codon 104 coded for a silent change (T104). In exon 6 of DLD1/HCT15, a C to A transversion caused a leucine-to-methionine substitution at residue 248 (L248M). In exon 5 of H69, a C to A transition was detected encoding a leucine to methionine substitution at residue 161 (L161M). No changes were found in the primary colon tumours. Since matching normal tissues do not exist for the cell lines to confirm somatic mutation events in these cases, we then screened independent normal DNAs to see if these differences exist in cells of noncancerous individuals. The exon 5 variant was found in one out of 47 normals, indicating that it is probably a rare polymorphism, but the exon 6 substitution was not seen in 54 normals, nor the silent exon 3 variant in 47 normals. There is thus a possibility that the L248M change is a true cancer-related mutation, although it is a conservative substitution and so may have little functional effect.

RT–PCR showed that *MBD3* expression is detectable in all the cell lines tested (data not shown), an observation confirmed for a selection of the colon cancer cell lines by Northern blot hybridisation of polyA+ RNA (Figure 2C), although expression in SW480 relative to *β*-actin appears lower than for other cell lines. We went on to test for methylation of the CpG island, but did not find evidence for methylation in any of the colon (including SW480) or lung cell lines (see Figure 2D for colon cancer cell lines, lung cancer cell lines data not shown). The method used to screen for methylation concentrated on the nine *Hpa*II/*Msp*I sites, all of which must be methylated to allow PCR and give a positive result after *Hpa*II digestion, and therefore gives a qualitative (total *vs* partial/no methylation) rather than quantitative assessment of methylation.

## DISCUSSION

Inactivating *LKB1/STK11* germline mutations in combination with loss of the wild-type allele by chromosomal loss or methylation are responsible for the development of hamartomatous polyps and adenocarcinomas in Peutz–Jeghers syndrome patients. *LKB1/STK11*, however, is rarely involved in sporadic colon cancer cases and at most 33% of NSCLC cases (Sanchez-Cespedes *et al*, 2002), leading us to consider the role of *MBD3* as an alternative tumour-suppressor gene on this location. This gene is tightly linked to *LKB1/STK11*, being only 500 kb away on chromosome 19p13.3. To investigate the role of *MBD3* in colon and lung tumorigenesis, we assayed for mutations, lack of expression and promoter methylation. We found two missense changes, one out of seven colon cancer cell lines and one out of 20 lung cancer cell lines, and none in colon primaries. Both changes were located outside the MBD, and one appears to be a naturally occurring rare polymorphism. The coincidence of the missense and silent mutations in DLD1/HCT15 may simply reflect the mismatch repair defect of these cell lines due to *MSH6* mutation. RT–PCR amplification and Northern blot hybridisation of the cell lines showed clear expression of the gene, while methylation-sensitive restriction enzyme/PCR analyses showed that none were fully methylated across the region tested. Hypermethylation of other genes involved in tumorigenesis usually shows methylation across the bulk of the associated CpG island. We would therefore have expected the nine CpG sites we tested of the *MBD3* CpG island (about 50% of the putative CpG island for *MBD3*) to have been methylated in a significant proportion of cells to give a detectable PCR result if this phenomenon had occurred. Our expression and methylation results are consistent with a lack of significant aberrant, tumour-associated silencing of the gene. In summary, we conclude that *MBD3* is not a major target of genetic or epigenetic alteration in colon and lung cancer.
